# Turnover of the actomyosin complex in zebrafish embryos directs geometric remodelling and the recruitment of lipid droplets

**DOI:** 10.1038/srep13915

**Published:** 2015-09-10

**Authors:** Asmita Dutta, Deepak Kumar Sinha

**Affiliations:** 1 Department of Biological Chemistry, Indian Association for the Cultivation of Science, Jadavpur, Kolkata; 700032, India

## Abstract

Lipid droplets (LDs), reservoirs of cholesterols and fats, are organelles that hydrolyse lipids in the cell. In zebrafish embryos, the actomyosin complex and filamentous microtubules control the periodic regulation of the LD geometry. Contrary to the existing hypothesis that LD transport involves the kinesin-microtubule system, we find that their recruitment to the blastodisc depends on the actomyosin turnover and is independent of the microtubules. For the first time we report the existence of two distinct states of LDs, an inactive and an active state, that occur periodically, coupled weakly to the cleavage cycles. LDs are bigger, more circular and more stable in the inactive state in which the geometry of the LDs is maintained by actomyosin as well as microtubules. The active state has smaller and irregularly shaped LDs that show shape fluctuations that are linked to actin depolymerization. Because most functions of LDs employ surface interactions, our findings on the LD geometry and its regulation bring new insights to the mechanisms associated with specific functions of LDs, such as their storage capacity for fats or proteins, lipolysis etc.

Lipid droplets (LDs), well known for their fat storage activities, are evolutionarily conserved organelles that exist in almost all eukaryotic cells^1^,^2^ as well as in a few prokaryotes^3^. Unlike other cell organelles that have a phospholipid bilayer enclosing an aqueous environment, LDs contain a neutral lipid core, rich in triglycerides (TGs) and sterol esters surrounded by a phospholipid monolayer^4^,^5^. The LD is studded with proteins on the droplet surface. Recent studies have identified a few LD-specific protein markers^6^. However, the presence of few proteins in the LD core has also been reported^7^,^8^. The diameter of LDs ranges from 0.1 to 5 μm in the case of non-adipocytes and can be more than 100 μm in the case of adipocytes^9^. LDs are mostly synthesized in the endoplasmic reticulum (ER), and some population of these LDs remain tethered to the ER even after synthesis^10^,^11^.

The role of LDs has been studied extensively at the cellular level. Apart from their canonical functions of storing fat and lipolysis, LDs behave as temporary storage platforms for hydrophobic proteins, such as α-synuclein, and serve as sites for their degradation^12^. Further literature reports indicate that the surface-to-volume ratio of a LD could influence the lipid metabolism rate^13^. This indicates the coupling of a LD’s geometry to its function. At the organismal level, experimental reports suggest the role of LDs in sequestering histone proteins transiently and acting as buffer systems to supply histones when required during the early embryonic development of *Drosophila*^14^. LDs are also known to prevent polyspermy in sea urchins^15^.

The droplet-like structures, also referred to as cortical granules, have been studied in zebrafish eggs, although their functional significance in the embryonic state, i.e., post-fertilization, has not been explored. The fertilization of zebrafish and medaka embryos is followed by ooplasmic segregation wherein the embryonic yolk separates from the ooplasm and the zygotic cell protrudes, forming the animal pole^16^,^17^. Prior to fertilization, the zebrafish zygote contains numerous cortical granules that undergo exocytosis and are believed to contribute to the remodelling of the zygote, leading to the formation of a yolk-blastodisc segregated zebrafish embryo^18^. Post-fertilization, a fraction of granular structures in the blastodisc has been reported to accumulate near the furrows. These structures co-localize with the adhesion protein β-catenin^19^. Therefore, it has been hypothesized that they could supply excess lipids and adhesion proteins, such as cadherins and β-catenin, to facilitate cytokinesis, leading to the formation of a new plasma membrane. Furthermore, the shape and size of LDs could be crucial factors that control the ratio of lipid-to-proteins in the newly formed plasma membrane. However, the origin and biophysical functions of LDs in the context of zebrafish embryonic development is poorly understood. In the course of ooplasmic segregation, how the LDs are recruited to the blastodisc or whether they are newly synthesized in the blastodisc remains unclear. Given the association of actin, myosin, microtubules with these LDs, their role in regulating the number, size and shape of LDs needs to be explored further.

## Results

### Lipid-rich granular structures are present in the zebrafish embryo

**Figure 1:**
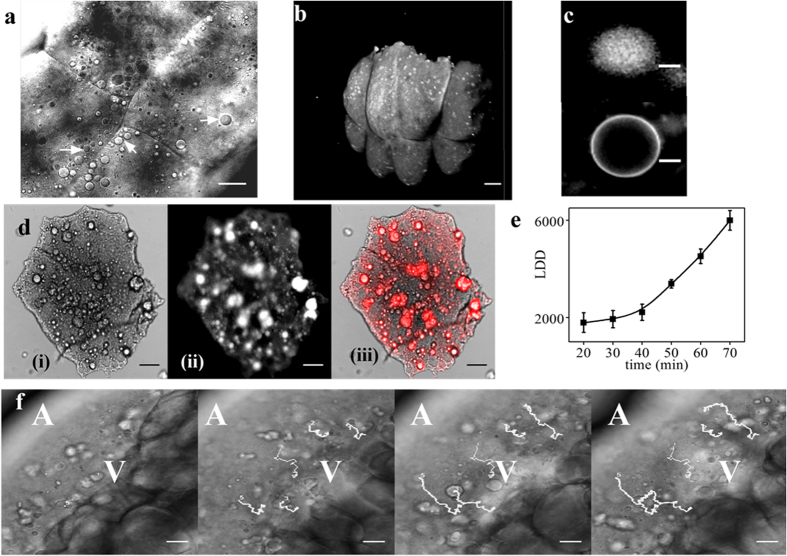
LDs are distributed cortically in the zebrafish blastodisc. (**a**) DIC image of zebrafish blastodisc at 4 cell stage (animal-pole view). Arrowheads point to the LDs. (**b**) Nile Red labeled 3D-rendered confocal image of blastodisc showing cortical distribution of LDs. (**c**) Nile Red stained images of LD (upper panel) and artificially synthesized giant vesicle (lower panel). (**d**) (**i**) DIC (**ii**) fluorescent (**iii**) merge images, of Nile Red stained de-yolked blastodisc showing co-localization of Nile Red signal (**ii**) with the granules of (**i**) conforming LD identity. (**e**) Mean LDD versus time averaged over three embryos, error bars are standard error of mean (SEM). (**f**) Time lapse images of blastodisc (lateral orientation) showing migration of LDs to cortical regions of the embryo. The white lines denote the trajectories of 5 representative LDs. ‘A’ and ‘V’ denote the animal and vegetal pole of the embryo. Scale bar 25 μm in (**a**) and (**f**), 8 μm in (**c**), 150 μm in (**b**) and 100 μm in (**d**).

We observed numerous granular structures in the blastodisc of the zebrafish embryos. A differential interference contrast (DIC) image of the embryo (animal pole/top view) at the 4-cell stage is shown in Fig. 1a. We noted a heterogeneous population of such granular structures present throughout the early developmental stages. These structures appeared to be present on the plasma membrane. We carried out scanning electron microscopy (SEM) imaging of the embryos to determine whether these granules are present on the membrane (Sup. Fig. S2). The SEM images ruled out this possibility. Visual inspection of DIC microscopy images showed that these structures could be either vesicles or another lipid-based object, such as a LD, present beneath the plasma membrane. To determine the nature of these droplets, we stained the embryos with Nile Red, which is a lipophilic dye well known for its use in visualizing intracellular LDs^20^,^21^. At a concentration of 300 nM, Nile Red stained both the yolk and blastodisc. The yolk is presumably rich in lipids and this makes it strongly fluorescent upon Nile Red treatment (Sup Fig. S3). Due to the high background fluorescence from the yolk, we did not observe any distinct granular fluorescent structure in the blastodisc. Therefore, we removed the yolk from the embryos to reduce the background fluorescence. Distinct fluorescent lipid structures in the blastodisc were then prominently visible (Fig. 1b,d).

### Granular structures in the blastodisc are LDs

The granules could be either bilayer vesicles or LDs or a mixed population of both. Thus, we compared the fluorescent images of artificially synthesized vesicles (similar size as the LDs), stained with the same concentration of Nile Red dye, with that of granules observed in the zebrafish blastodisc (Fig. 1c). As expected, the vesicles appeared as rings (Fig. 1c, lower panel), unlike the granules in the blastodisc, which were more like solid spheres (Fig. 1c, upper panel). Because the core of the LDs is rich in neutral lipids, we expected the lipophilic staining of the LDs to resemble the upper panel of Fig. 1c. Because all of the fluorescent granules in Fig. 1b resemble the upper panel of Fig. 1c, we conclude that all granular structures visible in the blastodisc are LDs and not giant bilayer lipid vesicles with an aqueous core. To further reaffirm the fraction of granules that are LDs, we performed a co-localization experiment with DIC and Nile Red stained images of de-yolked embryos. We found that all of the granules visible in the DIC channel are lipid-rich LDs (Fig. 1d).

### LDs are preferentially localized near the plasma membrane

To investigate the distribution of LDs in the blastodisc, we performed 3D-confocal imaging of the de-yolked embryos stained with Nile Red. Qualitative analysis, and 3D-image rendering revealed that the LDs were distributed uniformly in the blastodisc and preferentially localized near the cortex (Fig. 1b and Sup. Movie 1). Hence, we conclude that in zebrafish embryos, the LDs are present in the blastodisc and are cortically distributed beneath the plasma membrane.

### Lipid droplet density increases during early embryonic development

We observed an increase in the number of LDs in the blastodisc with embryonic development. Therefore, we quantified “lipid droplet number density” (LDD), the number of LDs per mm^2^ in live embryos. For this, we imaged the live embryos (animal-pole view, Fig. 1a) at 10-min intervals from the 1-cell stage (0–0.75 hour post-fertilization (hpf)) to the 4-cell stage (1–1.25 hpf). We counted all LDs having area equal to or more than 1.1 μm^2^. We observed a steady increase in LDD from 1789 ± 403 to 5993 ± 404 with the progression of embryonic development, as shown in Fig. 1e. This indicates that either the maternally synthesized LDs in yolk are transported to the blastodisc or the new LDs are synthesized in the blastodisc of the embryo.

### LDs are transported along the vegetal-animal pole axis in the blastodisc

To explore the increase in LDD with development, we performed time-lapse DIC imaging of the embryos along the lateral orientation for 1 hour (Fig. 1f and Sup. Movie 2). A continuous transport of LDs from the yolk-blastodisc interface towards the animal pole was observed. This suggests that most of the observable population of the LDs in the blastodisc is transported along the vegetal-animal pole axis towards the animal pole (Fig. 1f). We did not observe any LDs being transported from the bulk yolk region to the blastodisc.

### LDs exhibit active and inactive states during early development

**Figure 2:**
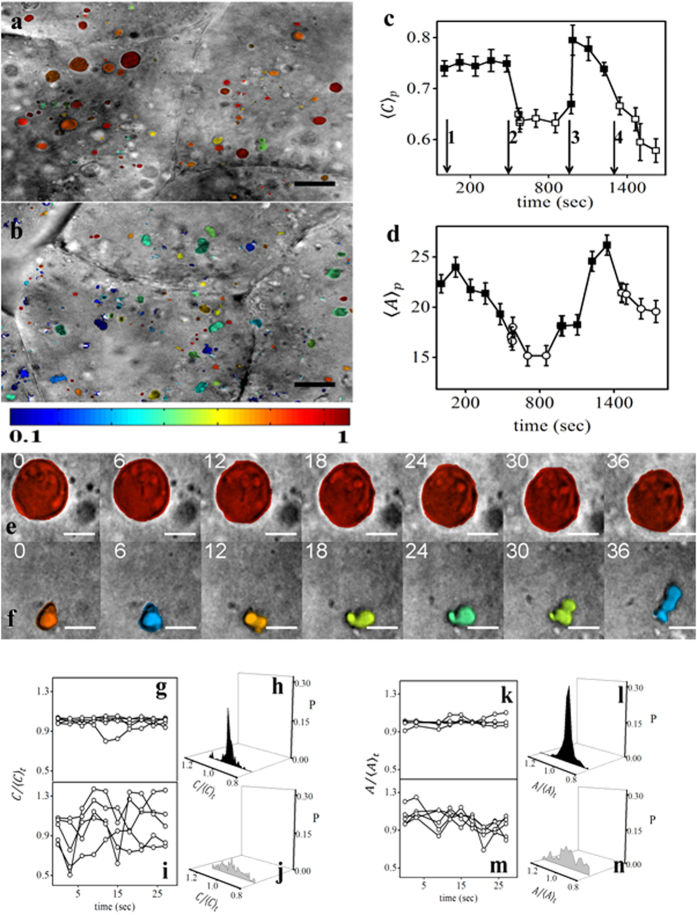
LDs periodically undergo active/inactive state transition. DIC images of zebrafish blastodisc at 1.25 hpf. The LDs have been pseudo-coloured based on their circularity values ranging from 0 to1 in (**a**) inactive state and (**b**) active state. Population average of (**c**) circularity, 〈*C*_*p*_〉 and (**d**) projected area, 〈*A*_*p*_〉 of LDs at different duration of embryonic development. Time lapse colour coded (circularity value) DIC image of representative LD in (**e**) inactive and (**f**) active state. Time stamps are depicted in seconds. (**g**,**i**) are the time traces of normalized circularity 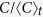 for 5 representative LDs in their inactive and active states respectively. (**h**,**j**) show the distribution of 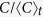 for 20 LDs in their inactive and active states respectively. (**k**,**m**) are the time traces of normalized projected area 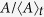 for 5 representative LDs in their inactive and active states respectively. (**l**,**n**) show the distribution of 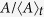 for 20 LDs in their inactive and active states respectively. The arrows in (**c**) denote the following: 1- initiation of 1^st^ furrow, 2- completion of 1^st^ furrow, 3- initiation of 2^nd^ furrow, 4- completion of 2^nd^ furrow. Open and closed symbols in (**c**,**d**) denote active and inactive states of the LDs, respectively. Scale bar 25 μm in (**a**,**b**), 8 μm in (**e,f**).

To understand the dynamics of LDs, we acquired time-lapse DIC images (animal-pole view) of the embryonic development at 3-sec intervals starting from the early single cell stage (10-min post-fertilization (mpf)) to 1–1.25 hpf. We observed that the LDs are dynamic and that they move in the blastodisc (Sup. Movie 3). We further observed that the LDs are very agile and undergo rapid shape fluctuations, except for the time interval that partially overlaps with the duration of cleavage. Based on this observation, we defined two states of the LDs in the embryo, an ‘active’ and an ‘inactive’ state that occur intermittently during early development. Because the cleavage occurs periodically, we studied the dependence of various geometric parameters, such as size and shape of LDs, on the active/inactive states. We compared the shapes of the LDs quantitatively during the two states. For this, we computed the circularity (see methods) of LDs using ImageJ. Figur 2a,b compares the circularity (scale 0.1–1) of the LDs during active and inactive states. We noted that most LDs are circular, as depicted by the red/orange colour code during the inactive state, whereas most LDs acquire an irregular shape as depicted by the blue/green colour code during the active state. This indicates that LDs are more circular in the inactive state. Additionally, we also noted that their size varies according to their active/inactive state. Figure 2c,d show that the population average of circularity 〈*C*_*p*_〉 and size 〈*A*_*p*_〉 of the LDs vary periodically, indicating that LD geometry is regulated periodically at the time scale of a few minutes. The periodic regulation of LD geometry is coupled to the active/inactive state of LDs as indicated by the closed and open symbols in Fig. 2c,d. Upon quantitation of the average speed of LDs, we did not observe such prominent periodic variation of speed coupled to the active/inactive state (Sup. Fig. S4). We believe this lack of observation is primarily due to the error in estimating the speed that is caused by the deformation of the blastodisc during cleavage (see supplementary note-1). We noted that the active/inactive states of the LDs are weakly linked to the cleavage formation during early embryogenesis, as indicated by arrows in Fig.2c.

### LDs’ shapes and sizes are more unstable during the active state

Having explored the statistical distribution of the shape and size of many LDs and their dependence on the active/inactive state, we next explored the temporal stability of individual LD geometry. For this, we analysed the time-lapse images of individual LDs at the time scale of a few seconds. Note that the shape fluctuation at this time scale is different from periodic regulation of LDs’ geometry (at few minutes time scale) associated with their active/inactive state. The shapes of LDs were more stable in the inactive than in the active state as depicted by the colour variation in Fig. 2e,f. We estimated the stability of the LDs’ geometry by quantifying the fluctuation of LDs’ *C* (circularity) and *A* (size) in specific states. To compare the shape and size fluctuations, the *C* and *A* values were normalized by time average circularity 〈*C*_*t*_〉 and size 〈*A*_*t*_〉, respectively. Observe an enhanced fluctuation of shape (*C*) and projected-area (*A*) for 5 representative LDs in the active state compared with those in the inactive state (Fig. 2g,i,k,m). To quantify the fluctuations, we determined the width of the respective histograms of normalized values of *C* and *A* for 20 LDs each in the inactive and active state. Figure 2h,j compared the distribution of 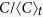 in inactive and active states respectively. Figure 2l,n compared 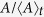 in the inactive and active states respectively. The wide distribution of both circularity and size of the LDs during the active state indicates that the shapes and sizes of LDs are comparatively more unstable during the active state.

Because LDs are transported to the cortical regions of the blastodisc leading to an increase in the LDD during early development, our next set of experiments were aimed at revealing the molecular players responsible for the recruitment of LDs to the blastodisc. There are two distinct mechanisms that can carry the LDs to the blastodisc cortex. Either the intermittent cytoplasmic streaming could carry the LDs along or the intra-cellular transport machinery, such as the actomyosin complex, or the microtubule/kinesin system could be utilized for the directed transport of LDs to the blastodisc. However, if the cytoplasmic streaming brought the LDs to the blastodisc, they need not be distributed cortically (Fig. 1b). Additionally, our observation that the LDs are distributed cortically, together with literature reports about biochemical analysis of LDs^22^, indicate the possibility of LDs being physically connected to the actin cytoskeleton, thus making cytoplasmic streaming irrelevant for their transport. Therefore, using pharmacological approaches, we disrupted the intra-cellular transport machinery to explore the molecular players responsible for the recruitment of LDs to the blastodisc.

### Recruitment of LDs to blastodisc is independent of microtubules

**Figure 3:**
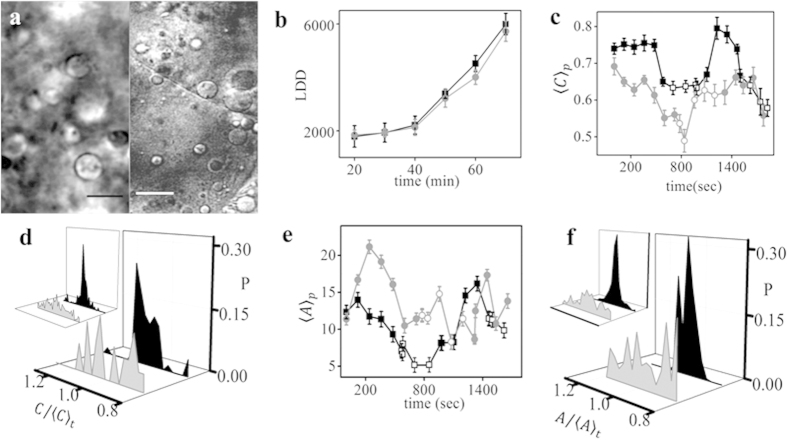
Effect of microtubule depolymerization on LD geometry, stability and recruitment in the blastodisc. (**a**) DIC image of zebrafish control embryo (left panel) and embryo treated with nocodazole (right panel). (**b**) Mean LDD versus developmental time for nocodazole treated and control embryos. Population average of (**c**) circularity, 〈*C*_*p*_〉 and (**e**) projected area, 〈*A*_*p*_〉 of LDs versus developmental time for control and nocodazole treated embryos. Distribution of (**d**) 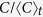 and (**f**) 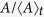 of LDs in their inactive (black) and active state (grey) for nocodazole treated embryos and control embryos (left inset). Nocodazole treated embryos are denoted by grey colour, round symbols and control embryos by black colour, square symbols while the open symbols denote active and closed symbols denote inactive state of the LDs in (**b**,**c**,**e**). All results are averaged over 3 independent measurements, error bars represent Standard Error of Mean (SEM). Distribution of shape and size have been estimated across 20 LDs. Area is in μm^2^ and the subscript ‘p’ and ‘t’ denotes average over population and time respectively. ‘P’ denotes probability.

We performed experiments to determine the role of microtubules in the recruitment of LDs to the blastodisc. We treated the embryos with nocodazole, which is an active microtubule depolymerizing agent^23^. The effect of microtubule depolymerization on the recruitment of LDs to the blastodisc was explored by performing similar experiments of live imaging of embryos at 10-min intervals for the same duration. Figure 3a compares the LD distribution in control and nocodazole-treated embryos. Similar to control embryos, the nocodazole-treated embryos also showed a significant increase of LDD from 1846 ± 182 to 5725 ± 375 with embryonic development (Fig. 3b). Various literature reports state the role of microtubule-dependent kinesin motors in the transport of LDs^24^. However, as expected, the inhibition of kinesin Eg-5 by monastrol did not prevent the increase in LDD with development. Thus, microtubule depolymerization and inhibition of kinesin Eg-5 did not produce any pronounced effect on the recruitment or distribution of LDs to the blastodisc.

### Periodic regulation of LDs’ shapes is dependent on microtubules

Unlike control embryos, the periodic regulation of LDs’ shape is lost significantly when the microtubules are disassembled. However, we still observed active and inactive states of the LDs (Sup. Movie 4). We noted a longer duration of the active and inactive states in nocodazole-treated embryos (Sup. Table-S1) compared with those of the control embryos. Interestingly, as shown in Fig. 3c, the average circularity of the LDs was always lower or the same as that of the control, indicating that the microtubules are crucial for maintaining the circular shape of LDs during the inactive state. As depicted in Fig. 3e, we did not observe any significant effect of microtubule disruption on the size distribution of LDs. Similar to the control embryos, we observed enhanced shape fluctuation during the active state (Fig. 3d). However, the projected area of the LDs did not show any significant change (Fig. 3f).

The filamentous actin structure provides shape to the cells and plays a major role in the short-range transport of small cargoes across cells^25^. Next, we explored the role of the actin cytoskeleton in the recruitment and maintenance of the LD geometry in the blastodisc. For this, we disrupted the equilibrium between ‘f’ and ‘g’ forms of actin.

### Reducing the levels of f-actin affects geometry, stability and recruitment of LDs

**Figure 4:**
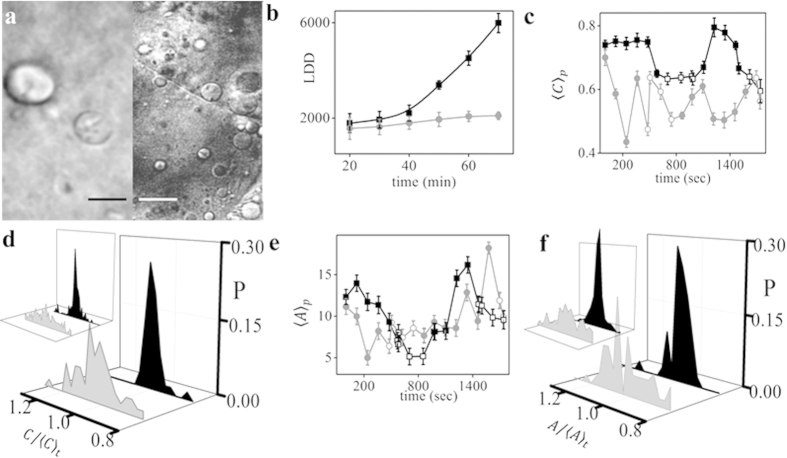
Effect of actin depolymerization on LD geometry, stability and recruitment to the blastodisc. (**a**) DIC image of zebrafish control embryo (left panel) and embryo treated with lat-B (right panel). (**b**) Mean LDD versus developmental time for lat-B treated and control embryos. Population average of (**c**) circularity, 〈*C*_*p*_〉 and (**e**) projected area, 〈*A*_*p*_〉 of LDs versus developmental time for control and lat-B treated embryos. Distribution of (**d**) 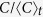 and (**f**) 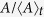 of LDs in their inactive (black) and active state (grey) for lat-B treated embryos and control embryos (left inset). Lat-B treated embryos are denoted by grey colour, round symbols and control embryos by black colour, square symbols while the open symbols denote active and closed symbols denote inactive state of the LDs in (**b**,**c**,**e**). All results are averaged over 3 independent measurements, error bars represent Standard Error of Mean (SEM). Distribution of shape and size have been estimated across 20 LDs. Area is in μm^2^ and the subscript ‘p’ and ‘t’ denotes average over population and time respectively. ‘P’ denotes probability.

Latrunculin B (lat-B) prevents the formation of f-actin, thereby shifting the equilibrium towards g-actin^25^. We treated the embryos with lat-B to reduce the f-actin level and performed live imaging of these embryos at 10-min intervals for 70 min. Figure 4a compares the LD distribution in control and lat-B-treated embryos. Figure 4b shows that the LDD did not increase significantly (1569 ± 459 to 2102 ± 160) in lat-B-treated embryos during the course of development. Therefore, actin depolymerization does have a pronounced effect on the recruitment and distribution of LDs in the blastodisc. Next, we analysed the shape and size of the LDs in lat-B-treated embryos. We found that on average, the LDs were significantly less circular in lat-B-treated embryos in both the active and inactive states than in the control embryos (Sup. Movie 5). We also found that the periodic regulation of average circularity 〈*C*_*p*_〉 was lost in these embryos (Fig. 4c). Furthermore, the reduction of the f-actin level reduced the stability of the shape and size of LDs in the active state, as seen in Fig. 4d,f. From Fig. 4e, we infer no significant change in the projected area 〈*A*_*p*_〉 of the LDs post-treatment with lat-B. However, the duration of the active and inactive states is affected, leading to a prolonged inactive state and a shorter active state.

### Stabilization of actin filaments affects geometry, stability and recruitment of LDs to blastodisc

**Figure 5:**
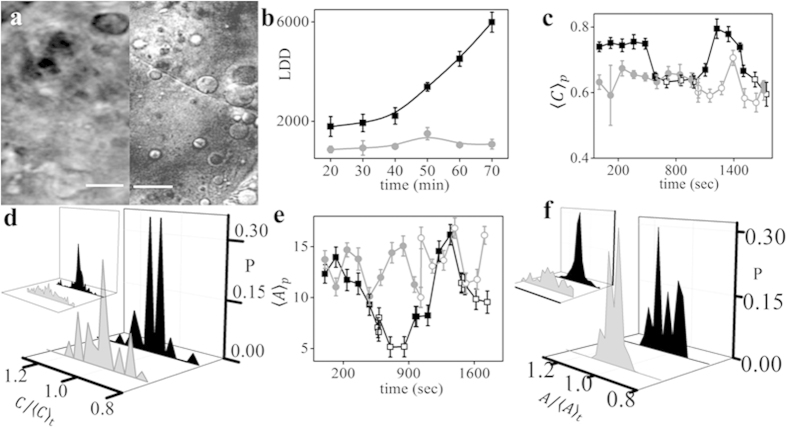
Effect of f-actin stabilization on LD geometry, stability and recruitment to the blastodisc. (**a**) DIC image of zebrafish control embryo (left panel) and embryo treated with phalloidin (right panel). (**b**) Mean LDD versus developmental time for phalloidin treated and control embryos. Population average of (**c**) circularity, 〈*C*_*p*_〉 and (**e**) projected area, 〈*A*_*p*_〉 of LDs versus developmental time for control and phalloidin treated embryos. Distribution of (**d**) 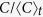 and (**f**) 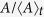 of LDs in their inactive (black) and active state (grey) for phalloidin treated embryos and control embryos (left inset). Phalloidin treated embryos are denoted by grey colour, round symbols and control embryos by black colour, square symbols while the open symbols denote active and closed symbols denote inactive state of the LDs in (**b**,**c**,**e**). All results are averaged over 3 independent measurements, error bars represent Standard Error of Mean (SEM). Distribution of shape and size have been estimated across 20 LDs. Area is in μm^2^ and the subscript ‘p’ and ‘t’ denotes average over population and time respectively. ‘P’ denotes probability.

We treated the embryos with a cell-permeable derivative of phalloidin, phalloidin oleate and performed live imaging of these embryos at 10-min intervals up to 1–1.25 hpf^26^,^27^. Figure 5a compares the LD distribution in control and phalloidin-treated embryos. Compared with control embryos, we observed a smaller number of LDs at 1–1.25 hpf (Fig. 5a) where the LDD did not increase significantly (861 ± 132 to 1076 ± 194) in phalloidin-treated embryos (Fig. 5b). Hence, we show that shifting the equilibrium to f-actin affects LD recruitment and distribution. We next quantified the shape and size of the LDs in phalloidin-treated embryos (Sup. Movie 6). The average circularity of the LDs in both the states in phalloidin-treated embryos was lower than that in the control embryos. We also noted that the periodic regulation of average circularity 〈*C*_*p*_〉 with time was lost in these embryos (Fig. 5c). We observed the same width of the distribution for shape and size during the active and inactive sates (Fig. 5d,f), indicating that the stabilization of f-actin resulted in reduced fluctuation of shape and size of the LDs in the active state. In other words, the LDs remain stable in terms of both shape and size even in the active state, unlike in the control embryos. From Fig. 5e, we conclude that the projected area 〈*A*_*p*_〉 of the LDs do not show any significant periodic variation. However, similar to lat-B, phalloidin-treatment too prolonged the inactive state and made the active state shorter.

The actin filaments are used as tracks by myosin-II motors for trafficking cargoes over short distances inside the cells^28^. Our next aim was to determine the role of Myosin-II motors, in the regulation of LD dynamics.

### Inhibition of non-muscle (NM) Myosin-II affects the geometry, stability and recruitment of LDs

**Figure 6:**
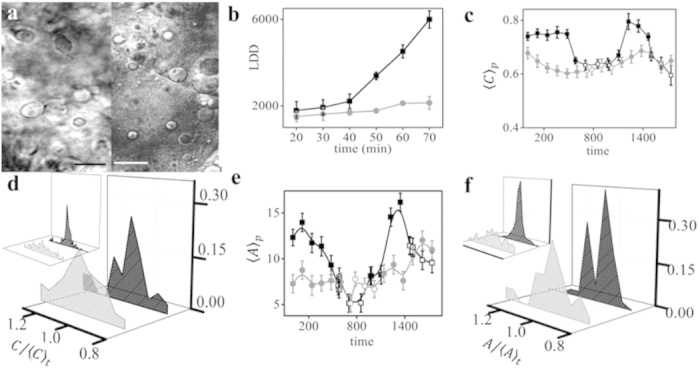
Inhibition of myosin-II on LD geometry, stability and recruitment to the blastodisc. (**a**) DIC image of zebrafish control embryo (left panel) and embryo treated with blebbbistatin (right panel). (**b**) Mean LDD versus developmental time for blebbbistatin treated and control embryos. Population average of (**c**) circularity, 〈*C*_*p*_〉 and (**e**) projected area, 〈*A*_*p*_〉 of LDs versus developmental time for control and blebbbistatin treated embryos. Distribution of (**d**) 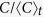 and (**f**) 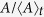 of LDs in their inactive (black) and active state (grey) for blebbbistatin treated embryos and control embryos (left inset). Blebbbistatin treated embryos are denoted by grey colour, round symbols and control embryos by black colour, square symbols while the open symbols denote active and closed symbols denote inactive state of the LDs in (**b**,**c**,**e**). All results are averaged over 3 independent measurements, error bars represent Standard Error of Mean (SEM). Distribution of shape and size have been estimated across 20 LDs. Area is in μm^2^ and the subscript ‘p’ and ‘t’ denotes average over population and time respectively. ‘P’ denotes probability.

Blebbistatin prevents the movement of myosin-II on actin^28^. We treated embryos with blebbistatin, and a similar live-imaging experiment was performed at 10-min intervals up to 1–1.25 hpf. Figure 6a compares the LD distribution in control and blebbistatin-treated embryos. Compared with control embryos, we observed a smaller number of LDs at 1–1.25 hpf (Fig. 6a) where the LDD did not increase significantly in blebbistatin-treated embryos ((1630 ± 359 to 2317 ± 324) (Fig. 6b). This shows that myosin-II hampers the recruitment of LDs to the blastodisc as well as its intracellular distribution. Next, we analysed the effect of myosin-II inhibition on the shape and size of the LDs (Sup. Movie 7). The average circularity of the LDs in blebbistatin-treated embryos was lower than that of control embryos in both the active and inactive states. As shown in Fig. 6c, the periodic regulation of circularity 〈*C*_*p*_〉 was also lost. Figure 6d,f, shows that blocking myosin-II does not produce any effect on the stability of the shape and size of the LDs. However, as shown in Fig. 6e, we noted that the average projected area 〈*A*_*p*_〉 of the LDs post-blebbistatin treatment was lower than that of the control. However, in contrast to other drug-treated conditions, the duration of the active and inactive states was not altered significantly in the myosin-II inhibited embryos (Sup. Table S1).

### Cortical recruitment of the LDs in the blastodisc is directed by actin turnover

**Figure 7:**
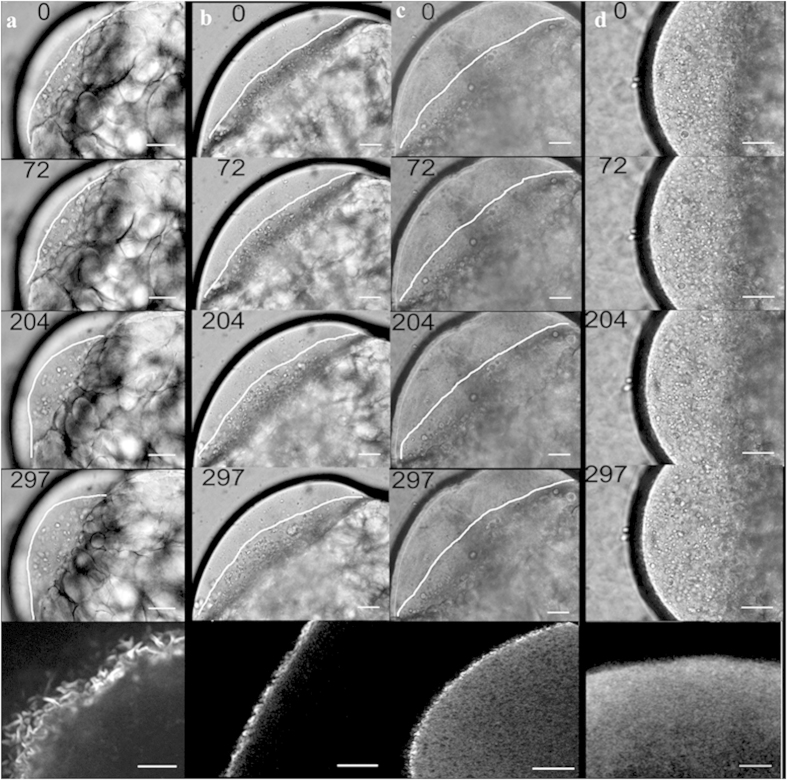
Time dependent distribution of LDs in the cortex of the blastodisc. Time lapse DIC images of the embryo (lateral orientation) showing the distribution of LDs in (**a**) control, (**b**) lat-B treated, (**c**) phalloidin oleate treated and (**d**) CK-666 treated embryos. The lowest panel in each column corresponds to the f-actin distribution in the blastodisc visualized upon staining by phalloidin-rhodamine. The white line marked in (**a**–**c**) encloses the region where most LDs are localized at that time point. Note that in (**d**), right from t = 0 time point, the LDs are distributed throughout the blastodisc cortex. Scale bars are 25 μm for all images.

To determine the source of the LDs, i.e., whether they are synthesized *de novo* or are transported from the yolk to animal pole, we carried out time-lapse experiments with embryos oriented laterally (side view) up to 1–1.25 hpf. In the case of control embryos, we observed directed migration of LDs from the yolk-blastodisc interface to the animal pole of the blastodisc (Figs 1e and 7a). However, upon lat-B treatment (Fig. 7b), we noted an accumulation of LDs at the yolk-blastodisc interface and these failed to migrate to the cortex. As expected, the treatment with lat-B reduced the thickness of the cortical f-actin band, whereas phalloidin oleate treatment resulted in a thicker band of cortical actin (shown in each corresponding lowest panel). The phalloidin treatment also caused severe accumulation of the LDs which failed to get distributed cortically (Fig. 7c). Next, we prevented the formation of actin branches by treatment with CK-666. As expected we observed a diffused distribution of f-actin (Fig. 7d, lowest panel). As shown in Fig. 7d, we observed that the LDs were no longer restricted to the cortical regions of the blastodisc but were spread throughout the entire blastodisc. Therefore, we report a significant role of actin turnover on the efficient distribution of the LDs to the blastodisc cortex. However, a similar experiment with blebbistatin did not show any LD accumulation and they were distributed throughout, although the overall number of LDs was low.

## Discussion

Recent experiments reveal that the LDs have a complex structure and are involved in various activities other than processing lipids^12^,^29^. However, whether the LD geometrical remodelling is related to their different functions is not yet known. We demonstrate that the LDs in zebrafish embryos are not passive balls of lipids but rather that they undergo geometrical remodelling during development, making them interesting organelles to explore the rich biophysical mechanism that allows for geometrical remodelling (shape and size fluctuation during active state) in a matter of a few seconds. Interestingly, we noted that the “fast geometrical remodelling” of LDs stops just before furrow formation and resumes after furrow completion (Fig. 2). During this period, the shape of the LDs is more persistent and, on average, their sizes are bigger. Thus, we define two states for LDs in the zebrafish embryo: 1) active state, when the LDs are motile and irregular in shape, which undergoes continuous rapid remodelling; and 2) inactive state, when the LDs are much less motile, have regular and round shape, and the round shape persists for longer. The LDs are much larger in the inactive state than in the active state. The inactive state partially overlaps with the time duration of furrow formation and cell division. The existence of two distinct states of LDs further suggests that their composition and mechanical properties could be different in distinct states. To the best of our knowledge, our study is the first to report the existence of two distinct states of LDs in any living system. Whether LDs undergo an active/inactive state transition that is partially coupled to cell division in other organisms or cell type is not known yet.

Here, we also report two novel findings related to LDs: 1) the dynamics of LD number density (LDD) and 2) the periodic regulation of LD geometry associated with the active/inactive state during early zebrafish embryonic development. The number of LDs in healthy cells are regulated within a range, whereas the accumulation of LDs beyond the pre-set value is associated with human diseases, such as obesity, diabetes, and atherosclerosis^30^,^31^,^32^. We observed that the LDD increases during early embryonic development (blastulation). Because the surface area of each blastomere keeps decreasing during blastulation, our result indicates the possibility that the embryo tries to maintain the absolute number of LDs per blastomere in a preset range (Sup. Fig. S5). Whether the new LDs in the blastodisc are synthesized *de novo* post-fertilization or the maternal pool of LDs present in the yolk is redistributed in the blastodisc, causing an increase in LDD, is not clear. The lateral visualization (Fig. 1e and Sup. Movie 2) of the embryo revealed that the LDs are transported actively from the yolk-blastodisc interface to the animal pole. However, we did not find any granular structure (LDs or vesicles) being transported from the bulk regions of the yolk to the blastodisc, suggesting that a large fraction of LDs in the blastodisc is synthesized post-fertilization. Note that new LDs may also form by the fission of existing LDs^33^,^34^.

**Figure 8:**
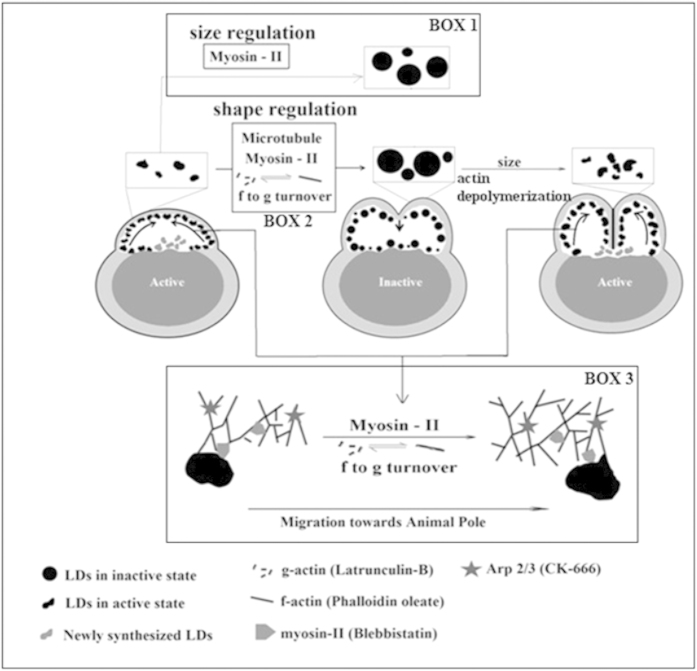
LDs are present in the zebrafish blastodisc. The recruitment of the LDs to the blastodisc is regulated by the actomyosin complex and not by the microtubules. The probable mechanism of recruitment of LDs by actomyosin complex is depicted in the Box1, note that this effect could be indirect. The LDs are dynamic and undergo periodic regulation of shape and size. Shape regulation of the LDs is maintained by the actomyosin complex and the microtubules. The myosin-II and g-actin plays role in size regulation of the LDs. LDs originate from the yolk-blastodisc interface and are distributed cortically in the blastodisc. LDs migrate to the animal pole and this migration is regulated by the actin turnover as well as myosin-II. The figure has been prepared by both AD and DKS.

The periodic regulation of LD geometry, both shape and size, could be associated with their differential function during the active and inactive states. We observed the accumulation of LDs near the site of furrow formation (Sup. Fig. S7). The furrow formation is rapid (initiation to maturation ~224 seconds), and a crude approximation indicates that the newly formed membrane would require 8–16% excess lipids along with a large amount of cell-cell adhesion proteins (Fig. 8, supplementary note 2 and Sup. Fig. S1). Because the LDs are loaded with lipids and proteins^35^,^36^, it is possible that they could supply the excess lipids and cell-junction-specific proteins, such as cadherins and catenins^19^, during the inactive state. By contrast, there is no such demand for lipids or adhesion proteins during the active state. Hence, we hypothesize that the LDs could accumulate the adhesion proteins and specific lipids to prepare themselves for the next inactive state that overlaps with the time of the cleavage.

LDs are distributed uniformly across the cortical blastodisc. The motility of LDs within the blastodisc is important to control their distribution. Thus, there must be elaborate transport machinery inside the blastodisc that maintains the uniformity of the LD distribution. Furthermore, we investigated the role of different molecular players involved in the recruitment of LDs to the cortical regions of the blastodisc and the periodic regulation of LD geometry using well-established pharmacological approaches.

Microtubule-based long-distance movements of LDs have been reported previously^37^. Additionally, the microtubule-associated motor proteins kinesin and dynein have also been found to be associated with LDs^37^,^38^,^39^,^40^. Interestingly, we found that depolymerization of microtubules did not affect the increase of LDD in the cortex, which indicates that the transportation of LDs to the cortex does not depend on the microtubules. Nevertheless, other effects not directly related to transport, such as regulation of LD shape and duration of both active and inactive states are affected (Sup. Table S1). A qualitative comparison of LD movement in control embryos with that of nocodazole-treated embryos indicates that the depolymerization of microtubules affects the motility of LDs in the active state (Sup. Fig. S6), yet we found that neither the cortical distribution of LDs nor the increase in LDD was dependent on microtubule-based transport machinery. This result indicates that the LDs do utilize the microtubule system for motility; however, in addition, there exists other parallel transport machinery that maintains the uniform distribution of LDs to the blastodisc cortex. Therefore, the actomyosin complex was investigated for its involvement in the recruitment of LDs to the cortical regions of the blastodisc.

Actin and actin-associated proteins, such as myosin, and Eps-15 homology domain protein, EHD2 have been reported to be associated with LDs^41^,^42^. We disrupted different components of the actomyosin complex to explore its connection with the dynamics of LDs. First, we shifted the equilibrium between ‘f’ and ‘g’ forms of the actin by treatment with lat-B (for more g-actin) or phalloidin (for more f-actin). Interestingly, irrespective of the direction of the shift (towards g-actin or f-actin), the recruitment of LDs to the cortical regions of the blastodisc is affected. The inhibition of myosin-II activity by blebbistatin also prevents the migration of LDs to the cortical regions of the blastodisc. Hence, we conclude that the turnover of the functionally active actomyosin complex is crucial for the directed migration of LDs. However the dependence of directed migration of LDs on the actomyosin turnover could be indirect. The mechanism that controls the directionality of the LD movement remains to be explored.

One of the most exciting findings of our work is the periodic regulation of the shape and size of LDs. The experiments conducted with treatments of different drugs revealed that both the actomyosin complex and the microtubule filaments jointly direct the periodic regulation of LDs geometry. Table 1 and Sup. Table S2 summarize the various geometric factors that are affected by different drugs. Although the size of LDs increases during the transition from the active state to the inactive state in control embryos, the inhibition of myosin-II activity prevented the growth of LDs during such a transition. Interestingly, the stabilization of f-actin failed to regulate the size of the LDs during the active state; as a result, we observed bigger LDs during the active state. The regulation of shape is even more complex. The stabilization of f-actin reduced the shape fluctuation of LDs during the active state, suggesting that the depolymerization of actin filaments is crucial for directing the shape fluctuation. Although the irregular shape of the LDs during the active state became more regular and round during the transition from the active state to the inactive state in the control embryos, any perturbation to the actomyosin complex or microtubule filaments prevented this shape regulation (Sup. Table S2, Sup. Fig. S9). This result indicates that the geometry of LDs is actively regulated and these geometrical parameters are controlled actively by different biomolecules.

## Methods

### Zebrafish culture and breeding

All animal experiments were carried out according to the guidelines approved by the Indian Association for the Cultivation of Science Animal Ethics Committee. Appropriate measures were taken to minimize pain or discomfort to animals. We purchased wild type strain of zebrafish *Danio rerio* from the local market and maintained them at a constant temperature of 28 °C. The fish were kept on a light cycle of 12 hours dark and 12 hours light. We cultured male and female fish in separate tanks under continuous water circulation system. For breeding, we moved the male and female fish to the breeding tanks at a ratio of 2:1 respectively with separators between them. These were kept overnight. Following morning, we removed the separators and allowed the fish to breed for 10 min and checked for embryos. Embryos were collected immediately and we transferred them to embryo media E3 (50 mM Nacl, 0.17 mM KCl, 0.33 mM CaCl_2_, 0.33 mM MgSO_4_) and we used these for further experimentations.

### Dechorionating and deyolking embryos

Freshly laid embryos were collected and these were dechorionated using the protocol of Link *et al.*, 2006^43^ with little modifications. In brief, for dechorionation, we soaked the embryos in 1 mg/ml solution of Pronase in E3 media for 2-3 minutes at 37 °C. As soon as one or two embryos fell out of the chorions, we added E3 to the dish. We carried on this procedure in a glass petri dish as post-dechorionation the embryos tend to stick to plasticware. All confocal and fluorescent images were taken for deyolked embryos post fixing them using Paraformaldehyde. For deyolking, 1 ml deyolking buffer (Fish Ringer buffer without Calcium: 55 mM NaCl, 1.8 mM KCl, 1.25 mM NaHCO3) was added to the embryos and kept for 1–2 min wit intermittent shaking till the yolk detached from the blastodisc.

### Imaging of Embryos

Post dechorionation, we embedded the embryos in 0.2% low melting agar. All embryos were agar embedded before carrying out all the experiments on imaging of the blastodisc for viewing LDs. LDD measurement of embryos were carried on by taking snapshots of the blastodisc of the embryos (top view) at an interval of every 10 min in control and all drug treated embryos. We used the upright microscope Olympus BX61 for all DIC images under a 40X objective. For determining the dynamics of LD, we carried on time lapse imaging with an interval of 3 sec between two successive frames.

To visualize filamentous actin in the zebrafish blastodisc, we stained the embryos with Phalloidin-Rhodamine. Embryos were collected and dechorionated. For control embryos, we fixed the embryos using 4% Paraformaldehyde for 4 hours and washed in PBS. These embryos were then incubated overnight in 66 nM Phalloidin followed by repeated PBS washes prior to imaging on a Nikon Eclipse Ti-E microscope at 60X magnification. For determining any effect of different drug treatments on the F-actin organization in the embryonic blastodisc, we treated the live embryos with respective drugs for 1 hour and then fixed and stained them similar to control embryos. For Nile red imaging, we fixed the embryos at different developmental stages using 4% paraformaldehyde and stained using 300 nM of nile red dye for 10 min followed by repeated washing. Imaging was done on under 20X objective using LeicaTCS SP8 confocal microscope.

### Drug treatments

We carried on all drug treatments on freshly laid, dechorionated embryos which were soaked in respective drugs immediately after embryo collection. Nocodazole, and Blebbistatin were each used at a concentration of 100 μM for 1–1.25 hpf. Lat-B concentration was kept to 2 μM. 1 μM of phalloidin oleate was used whereas CK666 was used at a concentration of 200 μM for the same duration. All drug concentrations were determined in accordance to previous reports.

### Image Processing and data analysis

We performed image processing to determine LD number, shape and size using ImageJ while color coding of LDs based on their circularity was done using Matlab. To determine LDD, we counted the number of visible LDs at each time point and divided it by the area of the blastodisc expressed in mm^2^. For the evaluation of the geometric parameters, namely shape and size of the LDs, we carried on the following steps: The edges of each LD were found out using ImageJ 1.47t and then they were converted into binary images using threshold adjustments. We then used the ImageJ Plugin ‘SIOX Segmentation’ to segment these LDs and remove the background interference. ‘Analyze Particles’ function of ImageJ was used to determine the area, perimeter of each LD. Using these we then calculated the shape factor of each LD by the ‘circularity’ parameter defined as:

Circularity (*C*) = √(Area/Perimeter^2^)

Circularity ranges from 0 to 1 where 1 corresponds to completely circular object. Next we expressed the area values in μm^2^ and used this data as the projected size of the LDs. We computed the velocity of the LDs by tracking individual LDs over nearly 20 frames using ImageJ Plugin MTrackJ.

## Additional Information

**How to cite this article**: Dutta, A. and Kumar Sinha, D. Turnover of the actomyosin complex in zebrafish embryos directs geometric remodelling and the recruitment of lipid droplets. *Sci. Rep.* **5**, 13915; doi: 10.1038/srep13915 (2015).

## Supplementary Material

Supplementary Information

## Figures and Tables

**Table 1:** Role of different cytoskeleton components on LD dynamics.

Property			Condition
V to A migration of LD	Yes		Control, Nocodazole
	No		Lat-B, Bleb, Phalloidin, CK666

Accumulation of LD	No		Control, CK666, nocodazole, bleb
	yes		Lat-B, Phalloidin

	During active state	During inactive state	
Size of LD	Small	Big	Control
	Small	**Small**	Bleb
	**Big**	Big	Phalloidin

Shape	Irregular	Circular	Control
	Irregular	**Irregular**	Nocodazole, Phalloidin, Lat-B, Bleb

	Less stable	More stable	Control
	**More stable**	More stable	Phalloidin

