# Supplementary material for: Turnover of the actomyosin complex in zebrafish embryos directs geometric remodelling and the recruitment of lipid droplets
Source: Sci Rep. 2015 Sep 10;5:13915. doi: 10.1038/srep13915 (PMC4650301; doi:10.1038/srep13915)
Supplement: Supplementary Information [file srep13915-s1.pdf]

Turnover of the actomyosin complex in zebrafish embryos directs geometric remodelling and the recruitment of lipid droplets

Asmita Dutta and Deepak Kumar Sinha*

Department of Biological Chemistry
Indian Association for the Cultivation of Science,
Jadavpur, Kolkata -700032, India
*bcdks@iacs.res.in

Supplementary Information

Note-1: Error in estimating velocity of LDs

Sup.Movie 3 shows large scale geometrical deformation of blastodisc cells associated furrow formation. LDs within the blastodisc exhibit motility independent of geometrical deformation of blastodisc cells. Thus the observed motion of LDs have two components 1) the motility caused by kinesin-microtubule or actomyosin complex 2) motility caused by geometrical deformation of blastodisc. Here we are interested in motility caused by molecular motors, since we cannot separate these two components of motility, the estimation of velocity is inaccurate.

Note-2: Estimation of excess lipids

We estimate the area of plasma membrane of the blastodisc at one cell stage by assuming it to be hemisphere of radius 200 microns. Area (A)= 2* pi *r^2 =25*10^4 Πm^2, where r is radius . The excess plasma membrane created during furrow formation is depicted by red lines in Fig-S1. Two bilayers of excess plasma membrane have dimensions of rectangle 100 × 200 Πm. Therefore A_{excess}=2*10^4 Πm^2 per bilayer.

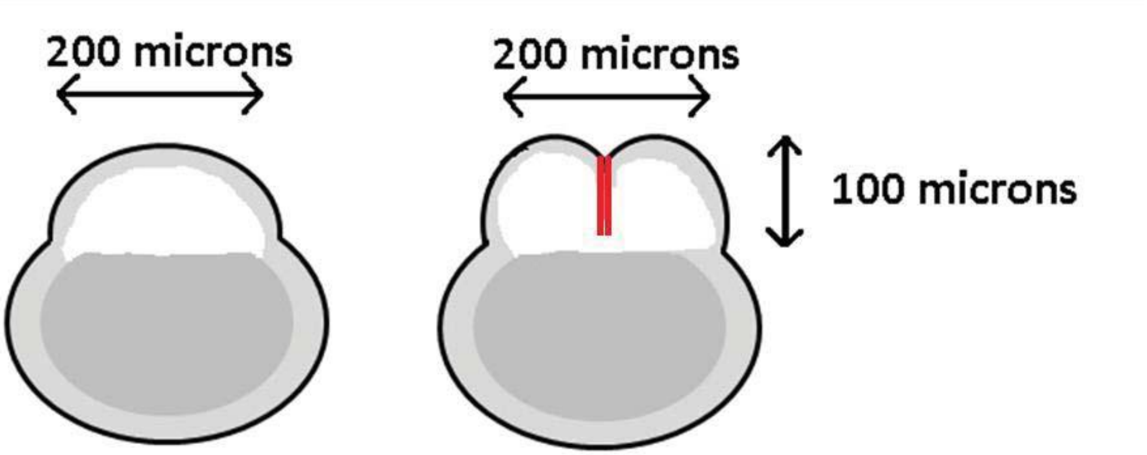

Figure S1. Approximate size of zebrafish blastodisc at one and two cell stages, the red lines indicate the new plasma membrane created during blastulation.

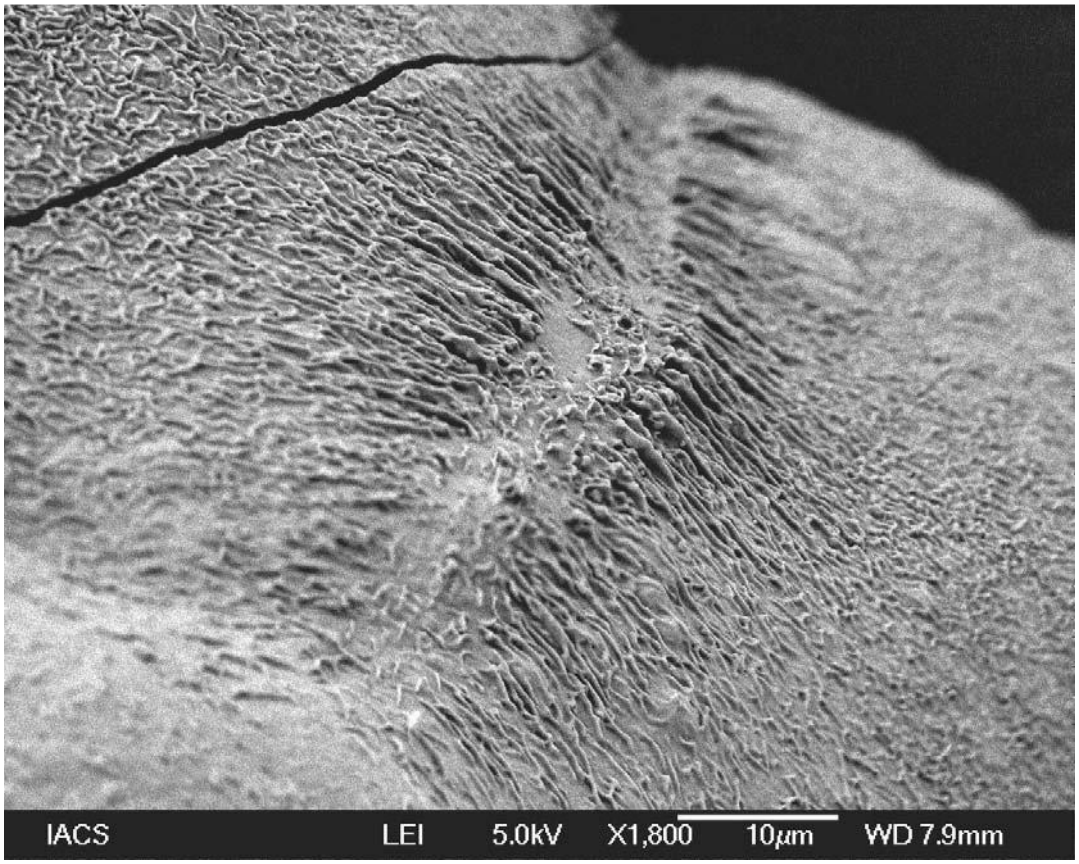

Figure S2. SEM image of zebrafish embryo. We fixed zebrafish embryos at 2 cell stage using 2.5% Glutaraldehyde for 2 hours. Next these were washed with PBS followed by treatment with Osmium tetraoxide for 1 hour. This was washed 3-4 times with 1X PBS. We then carried out serial dehydration of the embryos using increasing concentrations of ethanol. We stored the samples in a dessicator under vacuum conditions till imaging. The SEM image focused on the furrow of the embryo and also showed the complete absence of LDs on the embryo surface. This thereby provided evidence that the LDs were not present on the plasma membrane. A crack is visible on the sample which may be due to excess drying.

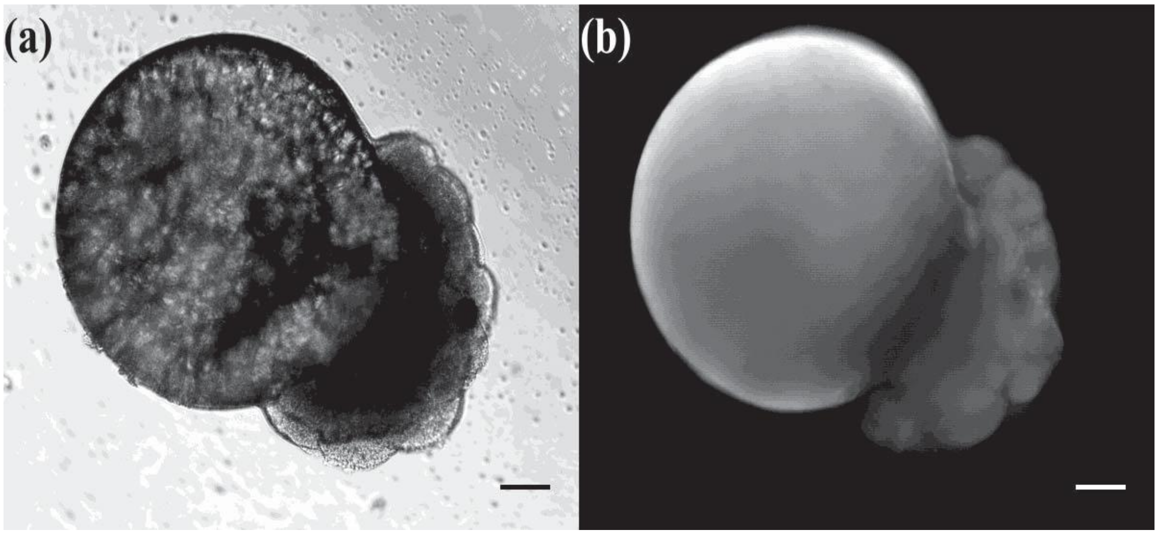

Figure S3. Strong fluorescence from lipid rich yolk. We collected zebrafish embryos and allowed them to grow to different stages. We then fixed these using 4% Paraformaldehyde (PFA) for 4 hours at room temperature. These fixed embryos were washed with PBS and dechorionated manually. This was followed by staining these with Nile Red for 10 min and subsequently washing them 3-4 times using 1X PBS to remove any excess dye. **(a)** DIC image of dechorionated embryo showing distinct yolk and blastodisc regions. **(b)** Fluorescent image of the same embryo. We found a very strong fluorescence of Nile red in the yolk of the embryo which made it difficult to visualize the blastodisc LDs without deyolking the embryo. Scale bar 150 μm.

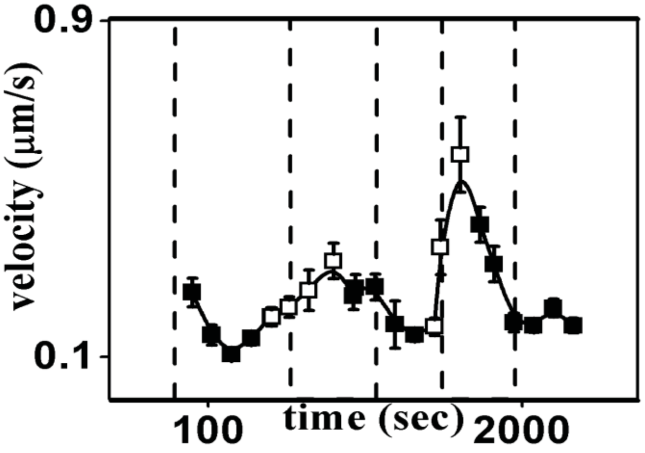

Figure S4. Average velocity of LDs in blastodisc during embryonic development. LDs undergo 'active' and 'inactive' states of motion wherein they have fast and slow movement respectively. We segmented individual LDs from entire image and tracked them up to 20 consecutive frames using ImageJ Plugin MTrackJ. The velocity of the LDs were calculated using the distance travelled by each LD in 20 frames and then averaged across all LDs. We notice a difference in average velocity of the LDs in active and inactive states. However, this difference was not significant. The dashed lines denote the duration of active and inactive states that we observed from the time lapse videos of control embryos.

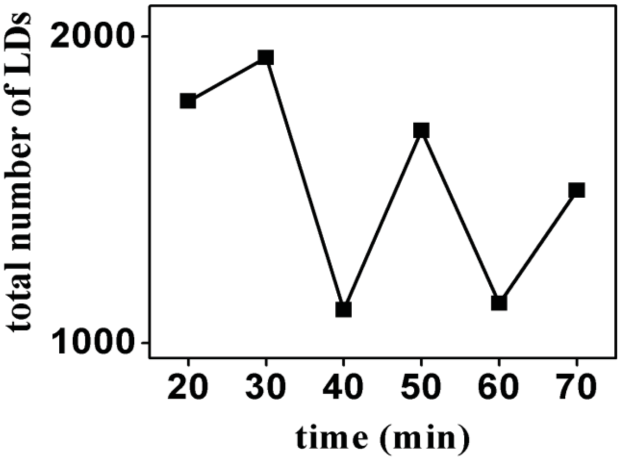

Figure S5. Number of LDs increases with time but is regulated by the cell number. We divided the LDDs by the cell area at each developmental stage. We notice that the embryo tries to maintain the average number of LDs per cell even thought the total number of LDs in the entire blastodisc increases with time.

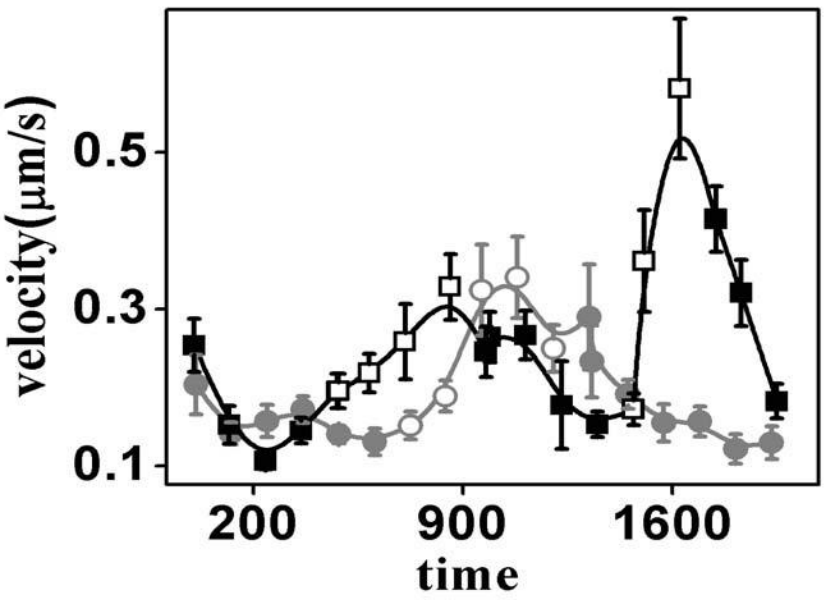

Figure S6. Effect of microtubule depolymerization on the motility of the LDs. We treated zebrafish embryos with nocodazole at a concentration of 100 μM and imaged after embedding in low melting agar. Top view time lapse imaging of the embryos revealed no apparent alteration in the recruitment and transport of LDs. Individual LDs were tracked using ImageJ Plugin MTrackJ and these were then used to calculate average velocities of the LDs across consecutive frames. In the above graph, closed symbols denote inactive and open symbols denote active state. The black line and square symbols show the velocity profile for control embryos whereas the grey line and round symbols depict the velocity profile of nocodazole treated embryos. Initially we notice a periodicity in the velocity of the LDs similar to control embryos at the earlier time points of the nocodazole treated embryos. However this is completely lost at later time points in the drug treated embryo.

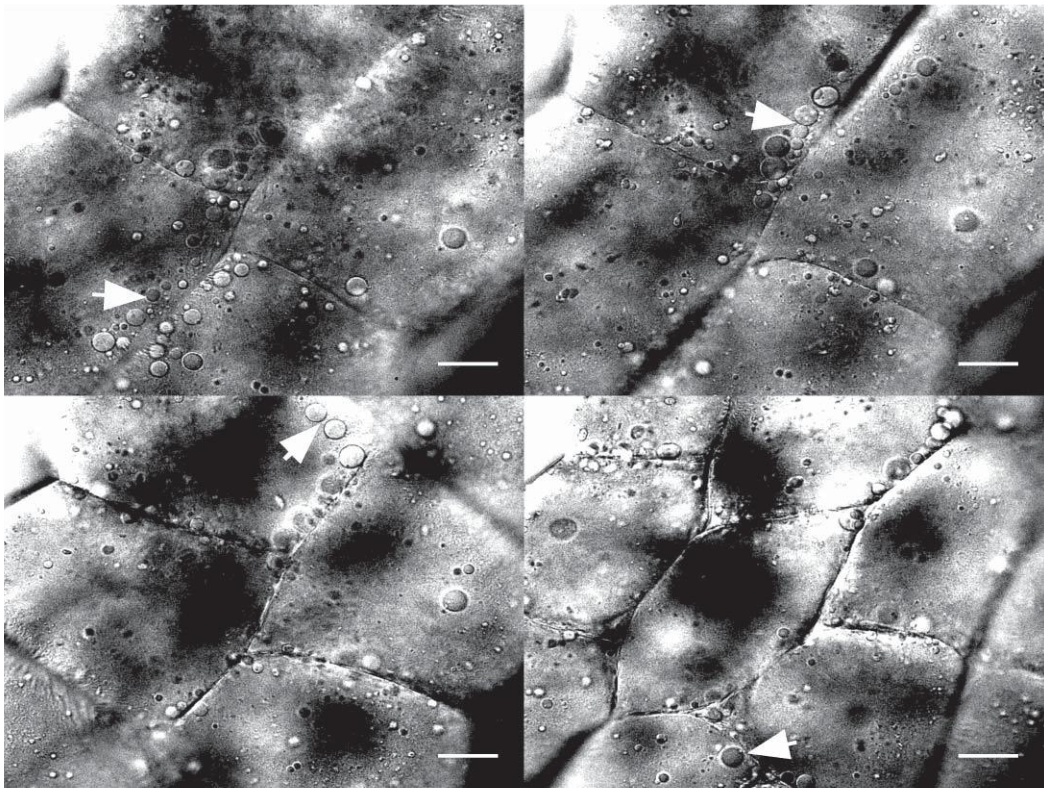

Figure S7. LDs accumulate at the sites of furrow onset and maturation prior to cleavage

Snapshots from time lapse video of control embryos. We noticed that LDs accumulate at the furrows indicating their probable role in supplying materials for new membrane formation. Scale bar 25 μm.

Table S1

Difference in the duration of active and inactive states of the LDs up on microtubule and acto-myosin complex perturbations in blastodisc of zebrafish embryo

	Duration of active state (sec)	Duration of inactive state (sec)
**Control**	347	466
**Nocodazole**	607	686
**Phalloidin**	680	905
**Latrunculin-B**	470	761
**Blebbistatin**	479	1037

Table S2. Qualitative comparison of overall effects of each drug compared to control embryo

Table S2. Qualitative comparison of overall effects of each drug to control embryo

Property / Drugs		Control			Nocodazole			Phalloidin			Latrunculin-B			Blebbistatin		
**LDD**		increases			increases			**No significant increase**			**No significant increase**			**No significant increase**		
**Existence of active and inactive state and duration (sec)**		Yes	347	466	yes	607	686	yes	680	905	yes	470	761	yes	479	1037
**Periodic regulation of**	**Shape**	Yes			**No**			**No**			**No**			**No**		
	**Size**	Yes			**No**			**No**			**No**			**No**		
	**Stability**	Yes			Yes			**No**			Yes			Yes		
**LDs in inactive state**	**Shape**	Circular			**Irregular**			**Irregular**			**Irregular**			Circular		
	**Size**	Bigger			**More Bigger**			Bigger			Smaller			Bigger		
	**Stability**	More stable			More stable			More stable			More stable			More stable		
**LDs in active state**	**Shape**	Irregular			Irregular			Irregular			Irregular			**Circular**		
	**Size**	Smaller			Smaller			Smaller			Smaller			**Bigger**		
	**Stability**	Less stable			Less stable			**more stable**			Less stable			Less stable		
**Accumulation of LD**		no			no			**Yes (++)**			**Yes (+)**			no		
